# Factors associated with mortality and functional status among neurocritical care adult patients: a prospective cohort study

**DOI:** 10.1186/2197-425X-3-S1-A985

**Published:** 2015-10-01

**Authors:** L Madeira, W Rutzen, L Tagliari, R Rosa, A Ascoli, JH Barth, E Oliveira, R Cremonese, T Tonietto, J Maccari, P Balzano, P de Leon, P Morandi, RPd Oliveira, C Teixeira

**Affiliations:** Moinhos de Vento Hospital, Porto Alegre, Brazil

## Introduction

Neurocritical care patients are at risk for the development of poor clinical outcomes during intensive care unit (ICU) stay. However, few data regarding the impact of specific factors on mortality and poor functional status in this population are available.

## Objectives

To evaluate factors associated with ICU mortality and poor functional status among neurocritical care adult patients.

## Methods

A unicenter prospective cohort study was conducted in a mixed medical-surgical ICU in southern Brazil between April 2008 and May 2013. All consecutive neurocritical care patients admitted during the study period were followed up. Stepwise Cox and logistic regressions were performed to identify factors associated with ICU mortality and poor functional status on the day of discharge from ICU (Rankin scale 4 or 5), respectively.

## Results

In total, 449 patients (47% men) were evaluated. The mean age and APACHE-II score were 63.8 years (SD 18.2) and 13.4 points (SD 7.4), respectively. The mean ICU length of stay was 8.8 days (SD 13.7). The overall ICU mortality was 8.4% (38 patients). The incidence of poor functional status on the day of discharge from ICU was 34.5% (142 patients). According to multiple Cox regression analysis, age (HR, 1.06; 95%CI, 1.03-1.09), need of mechanical ventilation during ICU stay (HR, 12.13; 95%CI, 2.86-51.42) and Hunt Hess scale at ICU admission for patients with subarachnoid hemorrhage (HR, 1.51; 95%CI, 1.20-1.89) were independently associated with mortality during ICU stay. After a multiple logistic regression was performed, Glasgow coma scale at ICU admission (OR, 0.79; 95%CI, 0.72-0.86), Apache-II score (OR, 1.05; 95%CI, 1.008-1.10), number of medical comorbidities (OR, 1.45; 95%CI, 1.13-1.86) and need of vasopressor during ICU stay (OR, 2.16; 95%CI, 1.06-4.40) were associated with poor functional status on the day of discharge from ICU among neurocritical adult patients.

## Conclusions

Neurological patients are a heterogeneous group of diseases, and were associated with substantial morbidity, mortality and cost; however, there is very little published work on this topic.
